# Extrachromosomal Circular DNA: An Emerging Potential Biomarker for Inflammatory Bowel Diseases?

**DOI:** 10.3390/genes15040414

**Published:** 2024-03-26

**Authors:** Valentina Petito, Federica Di Vincenzo, Lorenza Putignani, Maria T. Abreu, Birgitte Regenberg, Antonio Gasbarrini, Franco Scaldaferri

**Affiliations:** 1 Digestive Disease Center-CEMAD, Medicina Interna e Gastroenterologia, Fondazione Policlinico Universitario Gemelli IRCCS, Università Cattolica del Sacro Cuore, 00168 Rome, Italy; 2 UOS Microbiomica, UOC Microbiologia e Diagnostica di Immunologia, Dipartimento di Medicina Diagnostica e di Laboratorio, Ospedale Pediatrico “Bambino Gesù” IRCCS, 00146 Rome, Italy; 3 Division of Gastroenterology, Department of Medicine, University of Miami Miller School of Medicine, Miami, FL 33136, USA; 4 Department of Biology, Section for Ecology and Evolution, University of Copenhagen, 2100 Copenhagen, Denmark

**Keywords:** circular DNA, inflammatory bowel disease (IBD), ulcerative colitis (UC), Crohn’s disease (CD), colorectal cancer associated with colitis (CAC), extrachromosomal circular DNA (eccDNA)

## Abstract

Inflammatory bowel disease (IBD) comprising ulcerative colitis and Crohn’s disease is a chronic immune-mediated disease which affects the gastrointestinal tract with a relapsing and remitting course, causing lifelong morbidity. IBD pathogenesis is determined by multiple factors including genetics, immune and microbial factors, and environmental factors. Although therapy options are expanding, remission rates are unsatisfiable, and together with the disease course, response to therapy remains unpredictable. Therefore, the identification of biomarkers that are predictive for the disease course and response to therapy is a significant challenge. Extrachromosomal circular DNA (eccDNA) fragments exist in all tissue tested so far. These fragments, ranging in length from a few hundreds of base pairs to mega base pairs, have recently gained more interest due to technological advances. Until now, eccDNA has mainly been studied in relation to cancer due to its ability to act as an amplification site for oncogenes and drug resistance genes. However, eccDNA could also play an important role in inflammation, expressed both locally in the- involved tissue and at distant sites. Here, we review the current evidence on the molecular mechanisms of eccDNA and its role in inflammation and IBD. Additionally, the potential of eccDNA as a tissue or plasma marker for disease severity and/or response to therapy is evaluated.

## 1. Introduction

Inflammatory bowel disease (IBD) is a group of chronic multifactorial intestinal inflammatory disorders, including Crohn’s disease (CD) and ulcerative colitis (UC). These disorders arise primarily in genetically predisposed individuals and are influenced by a complex interplay of factors such as microbial dysbiosis, aberrant immune responses, and environmental triggers such as psychosocial stress, certain medications, dietary habits (e.g., Western diet), smoking, and the use and abuse of antibiotics [1]. The incidence and prevalence of IBD is increasing dramatically around the world, with a major increase in developing countries and young children [2]. In addition to the intestinal symptoms, these diseases have a serious impact on patients’ quality of life and their productivity [3].

Current medications include biological drugs like anti-tumor necrosis factor (anti-TNF), anti-integrins (vedolizumab), anti-IL12/23 (ustekinumab and risankizumab), and small molecules such as JAK inhibitors (tofacitinib, upadacitinib, and filgotinib). These therapies are associated with high costs, adverse events, and unfortunately rather modest long-term response and remission rates. In addition, almost no indicators are available to assist clinicians in selecting the best therapy for each patient [4]. Therefore, identifying predictive markers for response to therapy represents an urgent clinical need in IBD, allowing for successful treatment and subsequently reducing the risk of exposure to potentially harmful and ineffective therapies.

In the search for relevant biomarkers, cell-free DNA (cfDNA) could be considered. This type of DNA is defined as any DNA fragment free from its origin cell able to circulate freely in the bloodstream or within microvesicles. Previously, we have extensively described the main characteristics of different types of circulating DNAs [5]. Among cfDNA, in particular, extrachromosomal circular DNA (eccDNA) has gained interest as a potential biomarker. Emerging evidence from studying eccDNA might provide insights for understanding the etiopathogenetic and molecular mechanisms involved in several inflammatory and immune-mediated diseases and shed a light on new therapeutic strategies in clinical practice.

In this review, we therefore provide an overview of the recent literature on the relationship between eccDNA and inflammation, and the main molecular mechanisms involved. In addition, we also evaluate the potential use of eccDNA in IBD as a new type of diagnostic biomarker to classify the type of inflammation occurring in various human diseases and its potential to predict response to treatment.

## 2. Methods

PubMed (https://pubmed.ncbi.nlm.nih.gov, accessed on 2 July 2023) and Scopus (https://www.scopus.com, accessed on 2 July 2023) databases were searched using the following search string: (“IBD”, “ulcerative colitis”, “Crohn’s disease”) AND (“extrachromosomal DNA”, “circular DNA”, “circulating DNA”), of which only articles published between 2019 and 2023 were considered. The “AND” operator was used to create all possible combinations of selected terms. Only English research articles (original and review articles) were included, while articles without full-text reports were excluded.

## 3. Extrachromosomal Circular DNA

eccDNA is a term used to describe the full spectrum of eukaryotic extrachromosomal circular DNAs, which show a wide heterogeneity in size, ranging from hundreds to millions of base pairs, and have been classified in relation to their size and content, as previously described [6,7]. eccDNAs are double-stranded (ds), circular-shaped DNA fragments that exist independently from the chromosomal DNA in the nuclei of all eukaryotic species studied, such as drosophila [8], yeasts, and humans [9]. eccDNA is derived from nuclear chromosomal DNA and can harbor any type of genomic structure, including full-length and/or trunked genes, intergenic sequences, and repeated sequences. Moreover, eccDNA can be transcribed, giving rise to mRNA coding for full-length and/or truncated proteins and RNA with regulatory functions of gene expression (siRNA, miRNA, lncRNA, etc.) [10]. Moreover, eccDNA is common in both healthy and pathologic human tissue and blood, in sizes large enough to carry one or several complete genes [11]. Several models exist for how eccDNA is formed in human cells. The main mechanisms behind eccDNA production are errors during DNA repair processes (mismatch repair), hypoxia and chromosome shattering (chromothripsis), and errors during DNA replication and active DNA transcription (microdeletions) [6]. However, eccDNA can also be a product of cell apoptosis [9,12,13].

The presence of small circular fragments of extrachromosomal DNA, within the cellular nucleus, was already described in 1964 by Alix Bassel and Yasuo Hotta [14] during the study of Franklin Sthal’s theory. These “circles” were later identified in several types of human cancer cells [15] and recently also in healthy eukaryotic cells [11]. In addition, eccDNA has also been identified as a cell-free DNA (cfDNA) in human tissue [11,16], plasma [17], urine [18], and other biological fluids [19], and therefore, eccDNA could serve as a potential biomarker.

Notably, Moller et al. in 2018 revealed that large parts of the human genome can be detected in eccDNAs. This could potentially be explained by random mutational processes of repetitive sequences with extra space allowing for circularization [11]. In previous studies, short eccDNAs were detected with <2k bp (microDNAs), and even those as limited as only 100–200 bp were found in nuclei from muscle samples and leukocytes.

Previous findings have shown a high abundance of smaller cell-free eccDNAs in the plasma during healthy, inflammatory, and neoplastic conditions [16,17]. Interestingly, Pang et al. showed that healthy controls had significantly longer eccDNA sequences in plasma than patients with gouty arthritis [12].

## 4. Molecular Mechanisms of eccDNA and Its Role in Inflammatory Processes

eccDNA performs several functions within the eukaryotic cells, including gene amplification for both oncogenes and drug resistance genes in neoplastic cells [13,20], as well as having a role in intercellular heterogeneity and genomic instability [21,22], cellular aging [23], genetic compensation [24], intercellular communication [25], the restoration of telomere length (t-circles) [26,27], the production of short regulatory RNA, molecular sponges (microDNA), and immune regulation [10,28,29].

Cell-free circulating eccDNA could play a driving role in both systemic and tissue-specific inflammatory processes. For example, tissue-purified eccDNA, or synthetic circular DNA, but not their linear counterparts, have significant immunostimulatory activity, especially in the activation of the innate immune system. eccDNA has been shown to dramatically induce the production of type I Interferons (IFN-1α and IFN-1β), interleukin IL-6, and tumor necrosis factor (TNF-α) [29].

eccDNA activates innate immunity through the activation of several DNA-sensing pathways, including stimulator of interferon protein (STING) and a protein called absent in melanoma 2 (AIM2) [30].

A major source of eccDNA is mitochondrial DNA (mtDNA) which is released to the cytosol in response to apoptotic stimuli, next to cytochrome C. In the cytosol, this mtDNA could activate cyclic guanosine–adenosine monophosphate synthase (cGAS) to trigger inflammation [31]. Conversely, Wang et al. found that apoptosis could increase eccDNA generation, depending on apoptotic DNA fragmentation mediated by DNaseγ and ligation by DNA ligase 3, probably occurring in the late stage of apoptosis. These eccDNAs (<3 kb) were shown to have strong immunostimulatory activity mediated by the cGAS-STING pathway, depending on their circular shape rather than their sequence [29]. Extracellular vesicles (EVs) carrying double-stranded (ds) DNA, responsible for intercellular communication among intestinal cells, are significantly increased in murine colitis and active human CD, and positively correlated with disease activity via the activation of the STING pathway in macrophages [32].

Few hypotheses are available on the mechanisms of DNA damage inducing type I IFNs and other immune-regulatory cytokines [33,34]. DNA normally resides in the nucleus and mitochondria; hence, its presence in the cytoplasm serves as a DAMP to trigger immune responses. Cyclic guanosine monophosphate (GMP)–adenosine monophosphate (AMP) synthase (cGAS) detects DNA as a DAMP and induces type I IFNs and other cytokines [31,35,36,37]. In addition to NF-κB, MAPK and signal transducer and activator of transcription 6 (STAT6) are activated upon recognition of cytosolic dsDNA. Subsequently, STING stimulates autophagosome formation by facilitating the formation of puncta of microtubule-associated protein 1A/1B-light chain 3 (LC3) and autophagy protein 9a (ATG) [38]. In addition, beclin-1 (BECN1) inhibits the interaction between cGAS and dsDNA, thus restricting cGAMP formation in response to cytosolic dsDNA. The interplay between cGAS and BECN1 induces autophagy and the elimination of cytosolic dsDNA [39]. One of the main functions of autophagy is to eliminate and recycle cellular components, including cfDNA, in order to avoid inflammatory damage. Defects in the autophagy process enhance the recognition of cfDNA by various cytosolic pattern recognition receptors (PRRs) and enhance the response of the immune system [39].

IBD is characterized by defects in the autophagic machinery [40]. This is evident in the prevalence of polymorphisms within genes such as NOD2/CARD15, ATG16L1, and IRGM, which are linked to an increased disease risk. Therefore, it is reasonable to assert that eccDNA fulfils a major role in driving and enhancing intestinal and systemic inflammation in IBD.

In addition, IBD features an abnormal mucosal immune response against luminal bacterial products, including unmethylated double-stranded DNA, single-stranded RNA, and broad DAMPs. Due its ability to bind to TRL9, human endogenous eccDNA could also act as an endogenous DAMP, particularly due to the curvatures or U-shaped structures of eccDNA, and therefore could have an important pro-inflammatory role that is expressed both locally and systemically. The latter could be obtained by its ability to move in plasma within phospholipid vesicles or to move freely. Accordingly, eccDNA within vesicles can be assumed to be detected by innate immune cells, be phagocytosed, and activate intracellular pathways, such as cGAS-cGAMP-STING, which leads to the transcription of pro-inflammatory cytokine genes and thereby actively contributes to intestinal inflammation.

Chromothripsis, a complex phenomenon, involves the abrupt shattering of one or more chromosomes due to a sudden genomic instability event. Subsequent to this event, the shattered fragments undergo repair in a random order and orientation, potentially resulting in various outcomes, including DNA circularization [41,42,43]. In an inducible model of chromothripsis, the pharmacological inhibition of DNA ligase IV has been demonstrated to impede the reassembly of shattered fragments [44,45]. Additionally, sequence analysis of the junctions of rearranged chromosomes has indicated minimal or absent microhomology [46]. Consequently, levels of peripheral and oxidative DNA damage are elevated compared to those in healthy subjects, with distinct differences observed between patients with UC and CD patients [47].

Specifically, the study by Pereira et al. has highlighted that CD patients exhibit significantly higher levels of peripheral DNA damage compared to UC patients, whereas oxidative DNA damage is more pronounced in UC patients than in those with CD [48]. This variance may stem from the downregulation of DNA repair systems observed in UC patients, resulting in defects in the repair of 8-oxo-7,8-dihydro-2′-deoxyguanosine [8-oxo-dG]. This, in turn, leads to the accumulation of endogenously produced oxidized DNA bases, thereby increasing susceptibility to cancer development [49].

DNA transcription has the potential to modulate the mechanism of DNA circularization; however, the exact mechanism remains to be elucidated. Nonetheless, it has been proposed that the formation of R-loops during transcription could facilitate the direct repeats on the unpaired strand that form a loop which can subsequently be excised and ligated into a circular structure [50]. Transcription stress may render DNA more susceptible to damage [51], potentially leading to the generation of errors in DNA repair processes that could contribute to eccDNA formation. These observations establish a link between changes in the environmental conditions and eccDNA profiles, underscoring again the pivotal role of eccDNA in facilitating rapid adaptation to varying environments. Sequencing eccDNA in IBD holds promise for identifying the genes involved in the pathogenesis and mediation of the intestinal damage, as well as characterizing inflammation at the different disease stages.

Recently, apoptosis has emerged as a novel mechanism of biogenesis of cell-free eccDNA [29]. Apoptosis inducers have been shown to increase cell-free eccDNA generation, a process dependent on apoptotic oligonucleosomal DNA fragmentation mediated by DNaseγ, followed by ligation by DNA ligase 3, independent of both DNA ligase 1 and 4 [29]. Apoptosis is also a major source of cell-free DNA (cfDNA) [52,53], suggesting that cell-free eccDNA is a component of the cfDNA pool in the human body. Similarly, other mechanisms of cellular death, such as necrosis or pyroptosis [54], may also contribute to the pool of cfDNA. Pyroptosis, a pro-inflammatory cell death mechanism executed by gasdermin family proteins, including gasdermin B (GSDMB) and D (GSDMD), has been implicated in IBD susceptibility, regulating intestinal inflammation by promoting GSDMD-mediated pyroptosis [55].

Overall, these findings suggest the possibility of an increased amount of eccDNA resulting from inflammatory processes characterized by cell death, the accumulation of reactive oxygen species, and genomic instability [31,56,57,58,59,60].

## 5. Clinical Evidence regarding eccDNA in Inflammatory Bowel Disease

Only a few clinical publications have studied cfDNA in inflammatory bowel disease, and to the best of our knowledge, none have focused on eccDNA. In a pioneering study in 2003, Rauh et al. demonstrated the presence of cfDNA in the serum of UC patients, identifying a microsatellite alteration previously detected in mucosal cells from UC patients [61]. Building further on this, two different studies revealed a significant increase in cfDNA concentration in the plasma of mice with DSS-induced colitis compared to controls [62,63]. Recent findings further highlighted a progressive rise in total cfDNA levels in the plasma of mice with DSS-induced colitis [64,65], peaking at day seven of DSS administration and correlating with indicators of disease activity, such as neutrophil extracellular traps (NETs) [64,65]. Notably, the elevation in total plasma cfDNA did not correspond with an increase in cf-ncDNA or cf-mtDNA, indicating the potential involvement of other cfDNA types, such as eccDNA [65].

In contrast, while the total amount of cfDNA originating from colon tissue was increased only in the early stages of the disease, it was primarily associated with the increase in cf-ncDNA and cf-mtDNA subtypes [65]. These data highlight the importance of colonic inflammation in the early stage of the disease, subsequently leading to increased intestinal permeability and the release of cfDNA into systemic circulation, thereby contributing to the systemic immune activation.

Furthermore, IBD patients were shown to exhibit elevated levels of both ncDNA and mtDNA compared to healthy controls, suggesting that these types of DNAs could be used as biomarkers for IBD diagnosis, but not for monitoring disease activity [66]. Boyapati et al. additionally demonstrated that active IBD patients displayed higher levels of both circulating cfDNA and cell-free mtDNA, suggesting the use of mtDNA as a mechanistic biomarker of disease activity [67].

In the context of IBD, cfDNA can be generated by different types of programmed cell death, such as apoptosis, but also pyroptosis and NETosis [5,68,69]. As potential biomarkers, eccDNAs provide several advantages compared to linear DNAs. First, the circularly formed DNAs are resistant to exonuclease digestion due to their close circular structure, thereby being more stable than their linear counterparts [70]. Second, some eccDNAs found in the systemic circulation are much longer than their linear counterparts, which are normally 160–170 bp in size, facilitating their detection and dynamic monitoring [16]. Third, eccDNAs seem to have lineage specificity in some human cells, such as lymphoid cell lines, fibroblasts, and granulocytes [71]. Several studies have already demonstrated the potential application of circular extrachromosomal DNA elements in body fluids as biomarker candidates for the diagnosis, monitoring, and prognosis of various types of cancers and chronic kidney disease [18,72,73,74,75,76,77,78,79]. The growing evidence is suggestive of the potential of eccDNA as a biomarker in different types of diseases, including autoimmune or autoinflammatory diseases, such as IBD, particularly for monitoring disease progression and the severity of inflammation over time. Such insights may enable the implementation of aggressive therapeutic strategies for those patients with a high risk of severe inflammation. By serving as a mechanistic biomarker, eccDNA could aid in stratifying patients for relevant personalized therapeutic interventions, thereby advancing the field of personalized medicine in complex diseases such as IBD.

**Figure 1:**
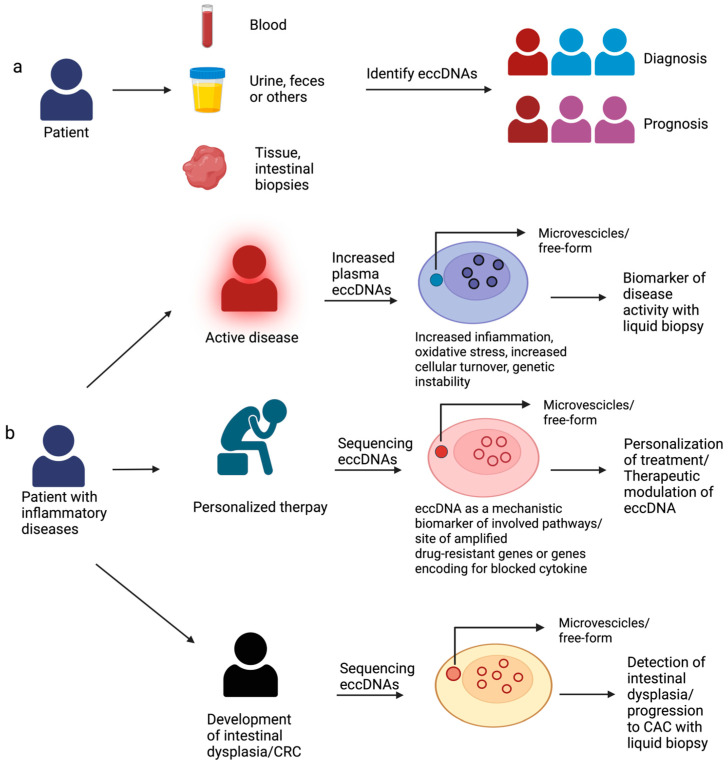
**eccDNAs as potential biomarkers in inflammatory bowel disease.** The clinical value of eccDNAs in IBD. (**a**) eccDNAs can be found in patient samples, such as blood, urine, feces, or intestinal biopsies. The identification of eccDNAs would be helpful in the diagnosis of IBD and prognosis. (**b**) The amount of eccDNAs in the intestinal mucosa and in the serum could correlate with the degree of intestinal inflammation, making it possible to assess the patient’s disease activity non-invasively. The sequencing of eccDNA will allow us to detect whether there are genes related to any non-response mechanisms, such as amplification of the targeted cytokine, enabling us to personalize the therapy and possibly even improve its efficacy due to therapeutic modulation of eccDNA. The sequencing of circulating eccDNA in serum could allow us to detect at an early stage the presence of genes (e.g., oncogenes) indicative of the development of intestinal dysplasia, without the need for continuous invasive testing.

Regarding IBD pathogenesis, eccDNA could play a major role in exacerbating mucosal inflammation by activating innate immunity and thereby establishing a vicious circle in affected patients. Consequently, the level of eccDNA may correlate with the disease severity and the extent of intestinal and systemic inflammation (Figure 1). This finding could revolutionize the management of IBD patients by offering a non-invasive means of tracking mucosal healing and assessing disease activity through the analysis of cell-free eccDNA. Such an approach would obviate the need for invasive endoscopic examinations, enhancing patient care and comfort. Additionally, there is a potential role for the use of cell-free eccDNA in the screening and detection of pre-IBD in high-risk groups, such as relatives of affected patients (Figure 1). Nuclear eccDNAs, capable of harboring entire genes or gene fragments, may encode modified proteins, as observed in cancer (16). Consequently, resistance to biological therapy in IBD may arise due to the amplification of specific genes or fragments within eccDNA molecules.

Finally, an intriguing novel clinical application (Figure 1) of eccDNA profiling lies in the surveillance for colorectal cancer associated with colitis (CAC). The profiling and sequencing of both plasma-circulating eccDNA and mucosal nuclear eccDNA could allow us to detect specific DNA mutations, oncogene amplification, or other eccDNA signatures which precede the development of detectable high-grade dysplasia, or which predict the evolution of low-grade dysplasia to CAC, thereby aiding in early intervention and management.

## 6. Conclusions and Future Perspectives

In conclusion, eccDNA profiling represents a revolutionary approach to monitor and guide therapeutic decisions in IBD. Rapid advancements in this field highlight the pivotal role of eccDNA in intracellular homeostasis and its pathological implications in various diseases. eccDNA as a biomarker in IBD may reduce the number of invasive endoscopic examinations that IBD patients must undergo over a lifelong period for the surveillance for CAC [80], allowing for the use of more rapid non-invasive tests, for example, with blood or even with fecal material. Large-scale prospective cohort studies are recommended to fully elucidate the diagnostic, prognostic, and predictive accuracy of eccDNA in immune-mediated disorders. Yet, the stability of eccDNA in tissue and blood holds promise for its integration as a prognostic biomarker in clinical practice.

Furthermore, understanding the mechanisms underlying eccDNA-mediated immune responses may unveil novel treatment strategies, ultimately paving the way for personalized medicine in IBD. Future research endeavors should aim to validate the potential of eccDNA as a biomarker and develop predictive models incorporating eccDNA analysis for personalized medicine in IBD.

## Data Availability

No new data were created or analyzed in this study. Data sharing is not applicable to this article.

## Abbreviations

8-oxo-dG = 8-oxo7,8-dihydro-2′-deoxyguanosine; AID = autoimmune diseases; AIM2 = protein called absent in melanoma 2; ALT = alternative lengthening of telomers; AMP = adenosine monophosphate; ATG = autophagy protein 9a; BECN1 = Beclin-1; CAC = colorectal cancer associated with colitis; CD = Crohn’s disease; cfDNA = cell-free DNA; cf-mtDNA = cell-free mitochondrial DNA; cf-ncDNA = cell-free nuclear DNA; cGAMP = cyclic GMP-AMP; cGAS = cyclic guanosine–adenosine monophosphate synthase; DAMP = damage-associated molecular pattern; DNaseγ = apoptotic DNA fragmentation; DSB = double-stranded breaks; dsDNA = double-stranded DNA; DSS = dextran sulfate sodium; eccDNA = extrachromosomal circular DNA; EV = extracellular vesicles; GMP = guanosine monophosphate; GSDMB = gasdermin B; GSDMD = gasdermin D; HR = homologous recombination; IBD = inflammatory bowel disease; IFN-α = interferon α; IFN-β = interferon β; IL-10 = interleukin 10; IL-17° = interleukin 17°; IL-17F = interleukin 17F; IL-18 = interleukin 18; IL-1β = interleukin 1 beta; IL-6 = interleukin 6; INF = interferon; LC3 = microtubule-associated protein 1A/1B-light chain 3; lncRNA = long non-coding RNA; MAPK = mitogen-activated protein kinase; MDP = muramyl dipeptide; miRNA = microRNA; MMEJ = microhomology-mediated end joining; MMR = mismatch repair; mtDNA = mitochondrial DNA; ncDNA = nuclear DNA; NETs = neutrophil extracellular traps; NF-κB = nuclear factor kappa B; NHEJ = non-homologous end joining; NIPT = non-invasive prenatal testing; PI3K = class III phosphatidylinositol 3-kinase; PRR = pattern recognition receptor; siRNA = small interfering RNA; spcDNA = small polydispersed circular DNA; STAT6 = signal transducer and activator of transcription 6; STING = stimulator of interferon genes; Th17 = T-helper 17; TLR-9 = toll-like receptor 9; TLRs = toll-like receptors; TNF = tumor necrosis factor; TNF-α = tumor necrosis factor α; UC = ulcerative colitis; ucf-eccDNAs = urinary cell-free extrachromosomal circular DNA; UTRs = untranslated regions.
